# Nac1 promotes self-renewal of embryonic stem cells through direct transcriptional regulation of c-Myc

**DOI:** 10.18632/oncotarget.17744

**Published:** 2017-05-10

**Authors:** Yan Ruan, Jianrong He, Wei Wu, Ping He, Yanping Tian, Lan Xiao, Gaoke Liu, Jiali Wang, Yuda Cheng, Shuo Zhang, Yi Yang, Jiaxiang Xiong, Ke Zhao, Ying Wan, He Huang, Junlei Zhang, Rui Jian

**Affiliations:** ^1^ Laboratory of Stem Cell and Developmental Biology, Department of Histology and Embryology, Third Military Medical University, Chongqing 400038, China; ^2^ Biomedical Analysis Center, Third Military Medical University, Chongqing 400038, China; ^3^ Department of Anesthesiology, Xinqiao Hospital, Third Military Medical University, Chongqing 400037, China; ^4^ Department of Cardiothoracic Surgery, Southwest Hospital, Third Military Medical University, Chongqing 400038, China; ^5^ Experimental Center of Basic Medicine, College of Basic Medical Sciences, Third Military Medical University, Chongqing 400038, China; ^6^ State Key Laboratory of Proteomics, Beijing Proteome Research Center, Beijing Institute of Radiation Medicine, Beijing 100850, China

**Keywords:** embryonic stem cells, Nac1, c-Myc, transcriptional regulation, self-renewal

## Abstract

The pluripotency transcriptional network in embryonic stem cells (ESCs) is composed of distinct functional units including the core and Myc units. It is hoped that dissection of the cellular functions and interconnections of network factors will aid our understanding of ESC and cancer biology. Proteomic and genomic approaches have identified Nac1 as a member of the core pluripotency network. However, previous studies have predominantly focused on the role of Nac1 in psychomotor stimulant response and cancer pathogenesis. In this study, we report that Nac1 is a self-renewal promoting factor, but is not required for maintaining pluripotency of ESCs. Loss of function of Nac1 in ESCs results in a reduced proliferation rate and an enhanced differentiation propensity. Nac1 overexpression promotes ESC proliferation and delays ESC differentiation in the absence of leukemia inhibitory factor (LIF). Furthermore, we demonstrated that Nac1 directly binds to the c-Myc promoter and regulates c-Myc transcription. The study also revealed that the function of Nac1 in promoting ESC self-renewal appears to be partially mediated by c-Myc. These findings establish a functional link between the core and c-Myc-centered networks and provide new insights into mechanisms of stemness regulation in ESCs and cancer.

## INTRODUCTION

Embryonic stem cells (ESCs) exhibit the capacity to undergo unlimited self-renewal and multilineage differentiation *in vitro* [1, 2]. These properties have made ESCs a powerful model for studying embryogenesis and a great source for regenerative medicine [3, 4]. Maintenance of ESC pluripotency depends on the coordination of transcription factors (TFs) [5–7], signaling pathways [8, 9], epigenetic modulations [10, 11] and microRNAs [12, 13]. Studies pertaining to the generation of induced pluripotent stem cells have revealed a central role for TFs in cell fate determination [14]. Oct4, Sox2 and Nanog together with additional TFs form the pluripotency transcriptional network. This network is crucial in maintaining an appropriate balance between ESC self-renewal and differentiation [7, 15, 16]. In order to fully understand the mechanisms that underpin pluripotency and early development of ESCs, it is imperative that we investigate the functions and regulatory relationships associated with factors involved in these regulatory networks.

The overall ESC gene transcription program can be subdivided into distinct units [17–19]. The Oct4/Nanog-centered core module includes genes that are mainly related to developmental and transcription-associated processes, whereas the c-Myc module contains targets which are predominantly involved in cellular metabolism, cell cycle, and protein synthesis pathways [6, 18, 20]. c-Myc has long been recognized as the key player to promote self-renewal of ESCs, by which the mechanisms may include potentiation of the autocrine Wnt/β-catenin signalling [21] and regulation of the mir-17-92 miRNA cluster [22, 23]. c-Myc has also been suggested to sustain pluripotency by repression of the developmental regulators directly or through upregulation of the Polycomb PRC2 complex [22, 24]. Moreover, the Myc-centered network activity has been proved to be shared by ESCs and cancer cells [18]. Nevertheless, the regulation of c-Myc in ESCs and the functional connections between core and Myc networks remain poorly understood.

Similar to c-Myc, Nucleus Accumbens-1 (Nac1) is also an oncogene which when overexpressed promotes cancer cell survival, growth, metastasis and resistance to chemotherapeutic drugs [25–29]. Previous studies have indicated that Nac1 physically interacts with both Oct4 and Nanog [30, 31], while also co-occupying targets with other key regulators in ESCs [17]. Therefore, Nac1 is regarded as a pluripotency-associated factor, which belongs to the core module. It has been reported that Nac1 knockdown (KD) induces ESC differentiation [31], whereas Nac1 knockout does not induce embryonic lethality or result in grossly noticeable morphological phenotypes in mice [32]. Following the use of integrative methodologies, Nac1 has been suggested to act as a lineage specifier that mediates mesendoderm (ME) and neuroectoderm (NE) cell fate selection [33]. However, the precise functions and mechanisms underlying the role of Nac1 in the regulation of ESC self-renewal and pluripotency are not well defined. The phenotypes of Nac1 overexpression (OE) in ESCs have not been addressed so far.

Here, we provide evidences that Nac1 is dispensable for pluripotency maintenance, but promotes self-renewal of ESCs through direct transcriptional regulation of c-Myc. Given the significant roles of Nac1 and c-Myc in cancer, our findings may implicate a potential mechanism underlying cancer biology.

## RESULTS

### Expression of Nac1 in preimplantation embryos, ESCs and during ESC differentiation

**Figure 1:**
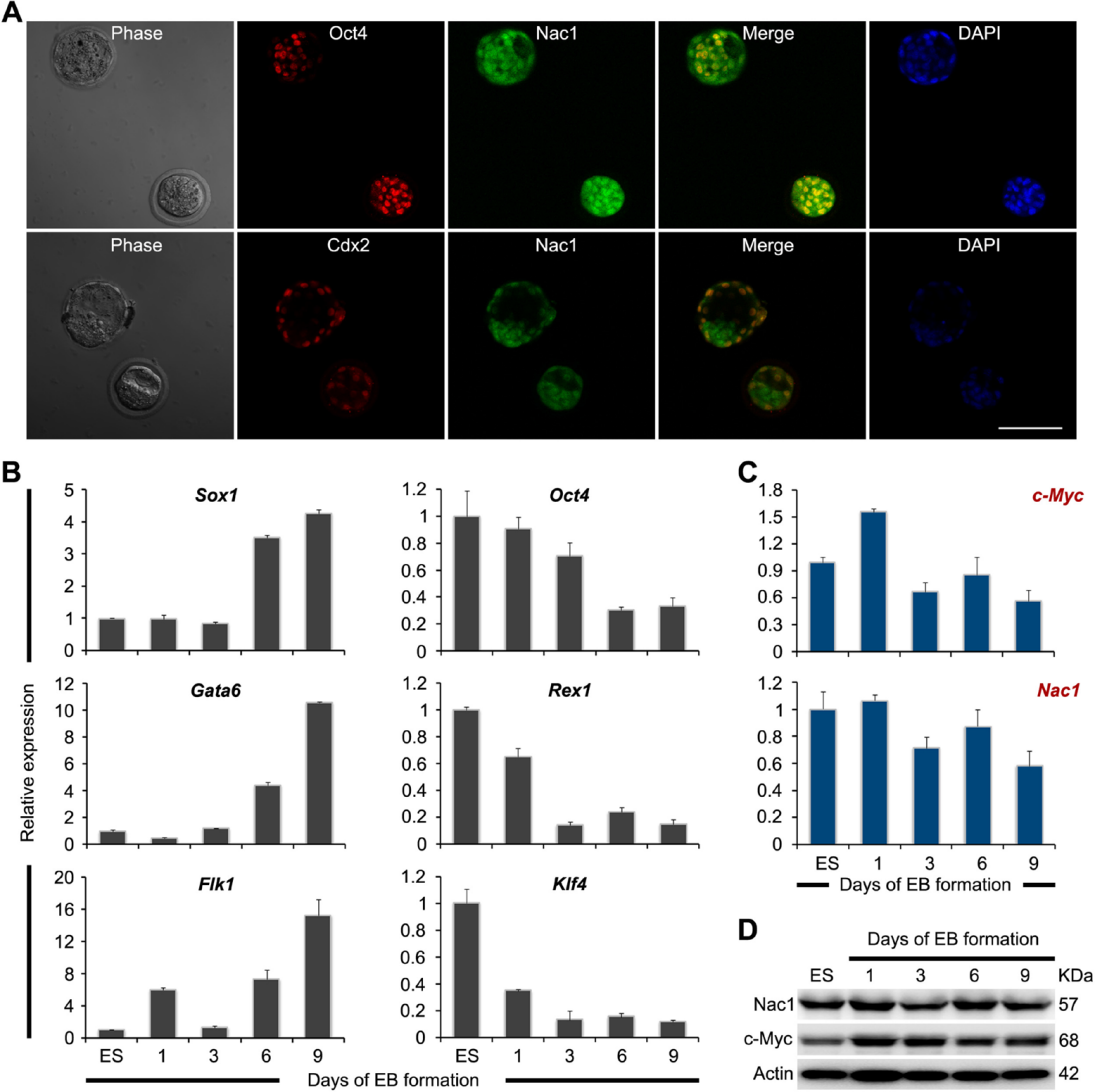
Expression pattern of Nac1 in preimplantation embryos, ESCs and during ESC differentiation (**A**) Co-immunofluorescence analysis of Oct4 (red) and Nac1 (green), or Cdx2 (red) and Nac1 (green) in preimplantation blastocysts. DAPI was used to visualize nuclei. Scale bar, 100 μm. (**B**) qRT-PCR analysis of germ layer and pluripotency marker expression levels during the course of EB formation. All data are normalized to *Gapdh* and shown relative to the mean of ESCs cultured in LIF (set at 1.0). Data are represented as means ± s.d.; *n* = 3. (**C**) qRT-PCR analysis of *c-Myc* and *Nac1* mRNA expression during the course of EB formation. All data are normalized to *Gapdh* and shown relative to the mean of ESCs cultured in LIF (set at 1.0). Data are represented as means ± s.d.; *n* = 3. (**D**) Immunoblot analysis of Nac1 and c-Myc proteins during the course of EB formation. β-Actin was used as an internal control.

To analyze expression patterns pertaining to Nac1 at the early stages of embryo development, we performed immunostaining on preimplantation mouse embryos. In accordance with previous reports [34], Oct4 was detected in the inner cell mass (ICM), while Cdx2 was only detected in the outer cells of the trophectoderm (TE). In contrast, Nac1 was found ubiquitously in both the ICM and TE (Figure 1A).

We subsequently characterized the expression profiles of Nac1 in undifferentiated ESCs and during embryoid body (EB) differentiation. Quantitative reverse transcriptase-PCR (qRT-PCR) analysis revealed that differentiation markers associated with the three germ layers were progressively induced after EB formation (Figure 1B), indicating efficient differentiation of ESCs. In contrast to the significant decrease of the core pluripotency genes *Oct4*, *Rex1* and *Klf4*, *Nac1* exhibited weak downregulation similar to that of *c-Myc* (Figure 1C). Western blot (WB) analysis confirmed that Nac1 was abundant in ESCs and reduced very slowly during EB formation (Figure 1D).

### Nac1 KD reduces the self-renewal efficiency of ESCs

**Figure 2:**
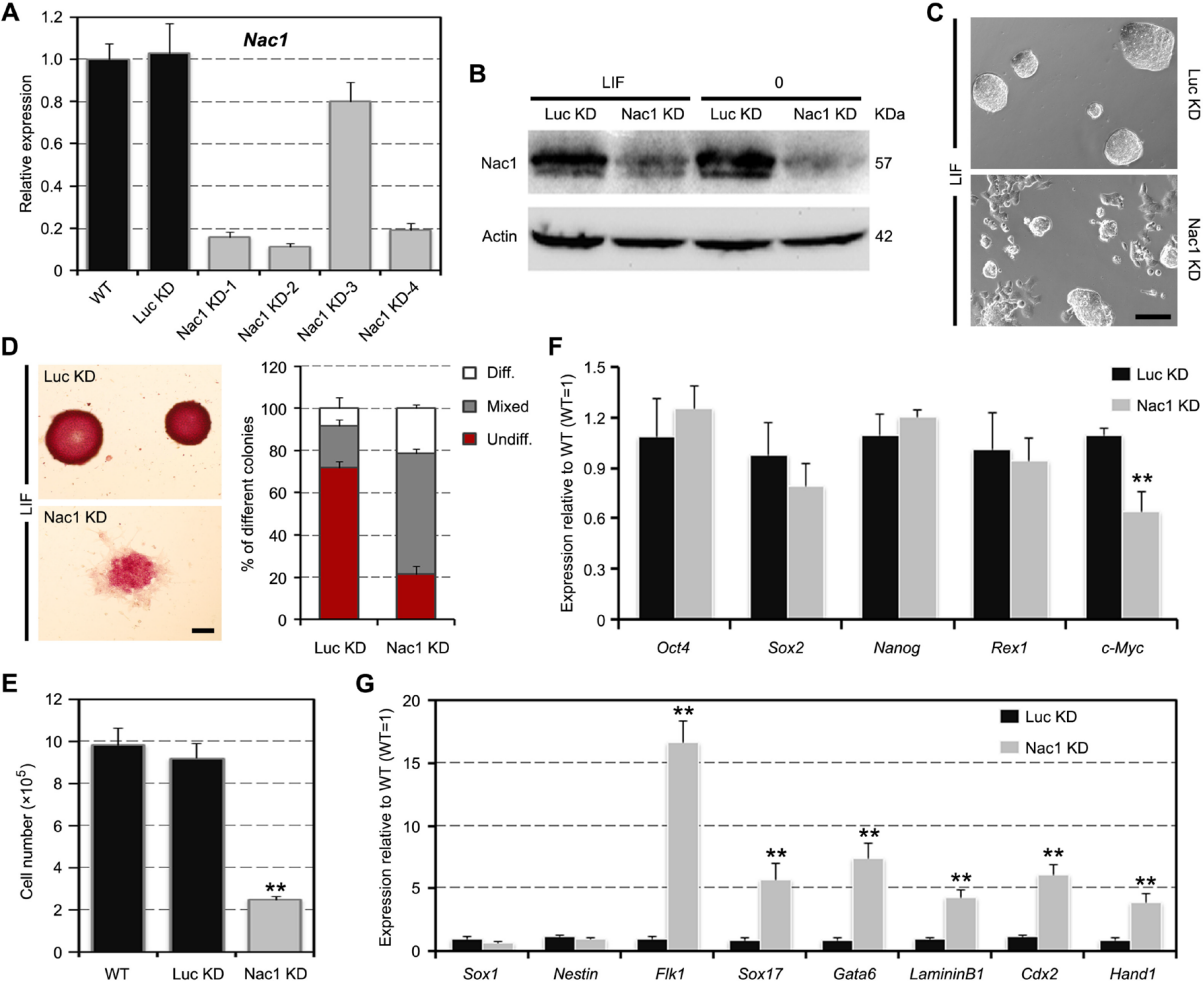
Nac1 KD reduces the self-renewal efficiency and induces an unstable pluripotent state in ESCs (**A**, **B**) qRT-PCR (A) and immunoblot (B) analyses after Nac1 KD. (**C**) Morphology of colonies formed by the indicated lines. Cells were grown with LIF for three passages after zeocin selection. Scale bar, 100 μm. (**D**) AP staining of colonies formed by plating the indicated cells at clonal density and culturing for 6 days with LIF (left). Scale bar, 100 μm. Percentage of colony types (undifferentiated, mixed, or differentiated) formed by cells is shown (right). (**E**) The indicated cells were cultured for 5 days with LIF and cell numbers were counted. (**F**, **G**) qRT-PCR analyses of pluripotency (F) and germ layer (G) marker expression levels in the indicated lines cultured with LIF. All data are normalized to *Gapdh* and shown relative to WT ESCs (set at 1.0). Data in (A) and (D–G) are represented as mean ± s.d.; *n* = 3. *p < 0.05; **p < 0.01. All *P*-values were calculated using Student's *t*-test.

To investigate the functional role of Nac1 in maintaining self-renewal, we used short hairpin RNA (shRNA) lentiviral vectors to stably KD Nac1 in ESCs. Four shRNAs targeting different regions of Nac1 complementary DNA were tested. Among them, the Nac1 KD-2 (named as “Nac1 KD” hereafter), which targets the 3’ untranslated region (UTR) of the *Nac1* gene, exhibited the most prominent silencing effect (Figure 2A, 2B). We found that the stable Nac1 KD ESCs could be easily established and continuously propagated in the presence of LIF. However, Nac1 KD cells exhibited a more differentiation-like morphology, formed less pure self-renewing alkaline phosphatase (AP)-positive colonies and grew more slowly than the luciferase KD (Luc KD, serving as a negative control) cells (Figure 2C–2E). qRT-PCR showed a modest downregulation of c-Myc and normal expression of the core module pluripotency-associated transcripts in Nac1 KD cells (Figure 2F). Meanwhile, we observed significant upregulation of ME (*Flk1*, *Sox17*, *Gata6* and *LamininB1*) and TE (*Cdx2* and *Hand1*) markers, but not of the NE (*Sox1* and *Nestin*) markers (Figure 2G). To further validate the specific Nac1-mediated effects, we repeated the experiments using Nac1 KD-1 and -4 ESC lines, and similar results were obtained (data not shown). These results suggest that Nac1 KD reduces the self-renewal efficiency and enhances the differentiation propensity of ESCs.

### Nac1 KD ESCs still remain pluripotency

**Figure 3:**
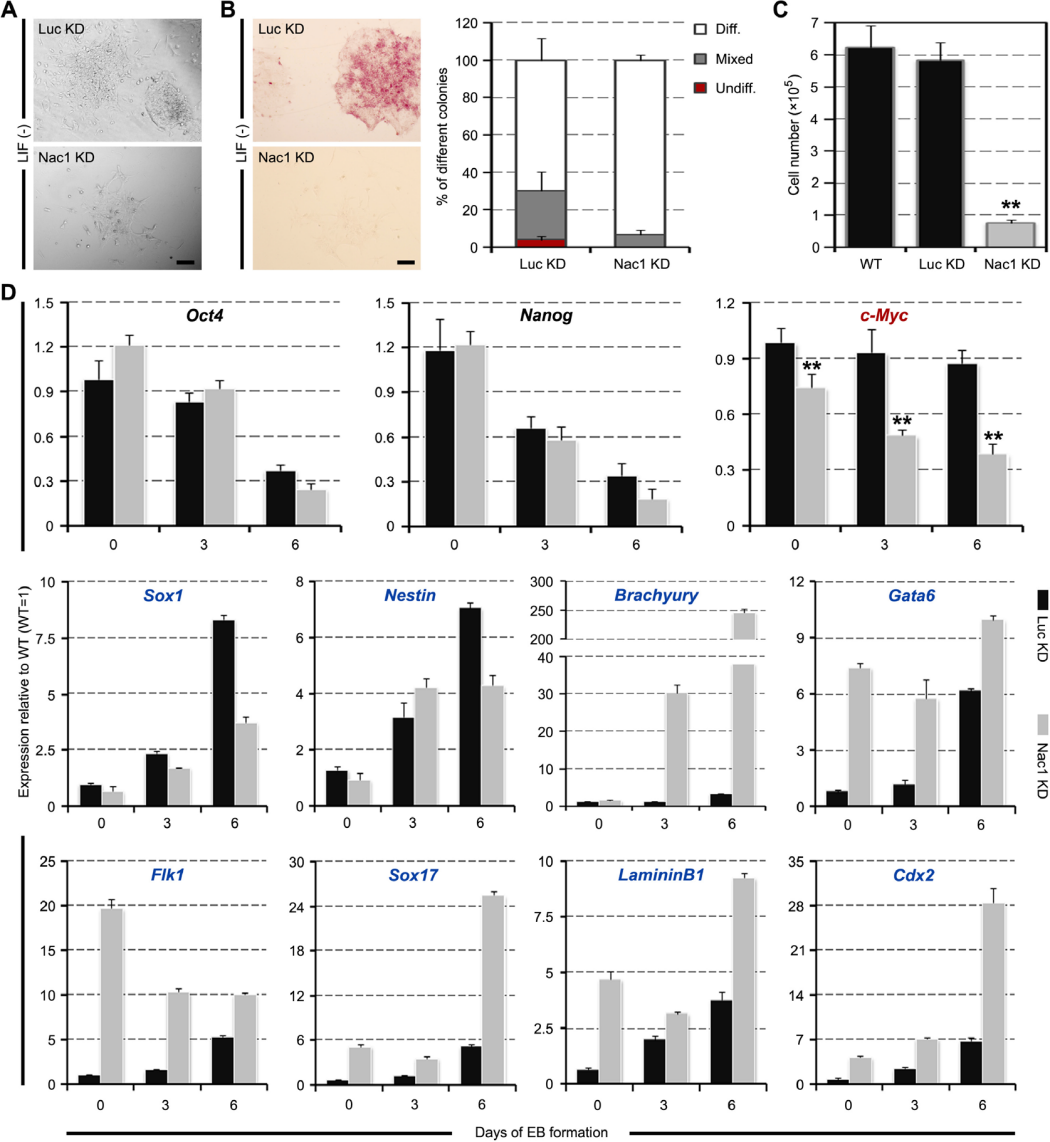
Multilineage differentiation of Nac1 KD ESCs (**A**) Morphology of colonies formed by the indicated lines. Cells were grown without LIF for 6 days. Scale bar, 100 μm. (**B**) AP staining of colonies formed by plating the indicated cells at clonal density and culturing for 6 days without LIF (left). Scale bar, 100 μm. Percentage of colony types formed by cells is shown (right). (**C**) The indicated cells were cultured for 5 days without LIF and cell numbers were counted. (**D**) qRT-PCR analysis of gene expression in the indicated lines after 0, 3 and 6 days of EB differentiation. All data are normalized to *Gapdh* and shown relative to WT ESCs (set at 1.0). Data in (B–D) are represented as mean ± s.d.; *n* = 3. *p < 0.05; **p < 0.01. All *P*-values were calculated using Student's *t*-test.

To assess the cellular characteristics of Nac1 KD cells during ESC differentiation, we plated Nac1 KD ESCs on gelatin at clonal density and cultured in the absence of LIF. Compared to Luc KD cells, Nac1 KD cells showed a more flattened morphology, an increased ratio of fully differentiated colonies and smaller colony size (Figure 3A, 3B). Accordingly, the growth rate of Nac1 KD cells was significantly lower than that of the control cells (Figure 3C), indicating that Nac1 is also required for the proliferation of differentiating ESCs. To address whether Nac1 deficiency affects the pluripotency of ESCs, we measured marker gene expression in developing EBs derived from Nac1 KD ESCs. As shown in Figure 3D, along with a gradual decline in the expression of pluripotency-associated genes, all embryonic germ layer markers were induced, indicating that Nac1 KD cells still retain the multilineage differentiation potential. In contrast to the slower upregulation of NE markers, Nac1 KD cells expressed a higher level of ME and TE markers than control cells, suggesting that Nac1 silencing may accelerate ESC differentiation to these lineages. In addition, we found that the expression of *c-Myc* was consistently inhibited in Nac1 KD cells during EB differentiation (Figure 3D).

### Nac1 OE delays ESC differentiation

**Figure 4:**
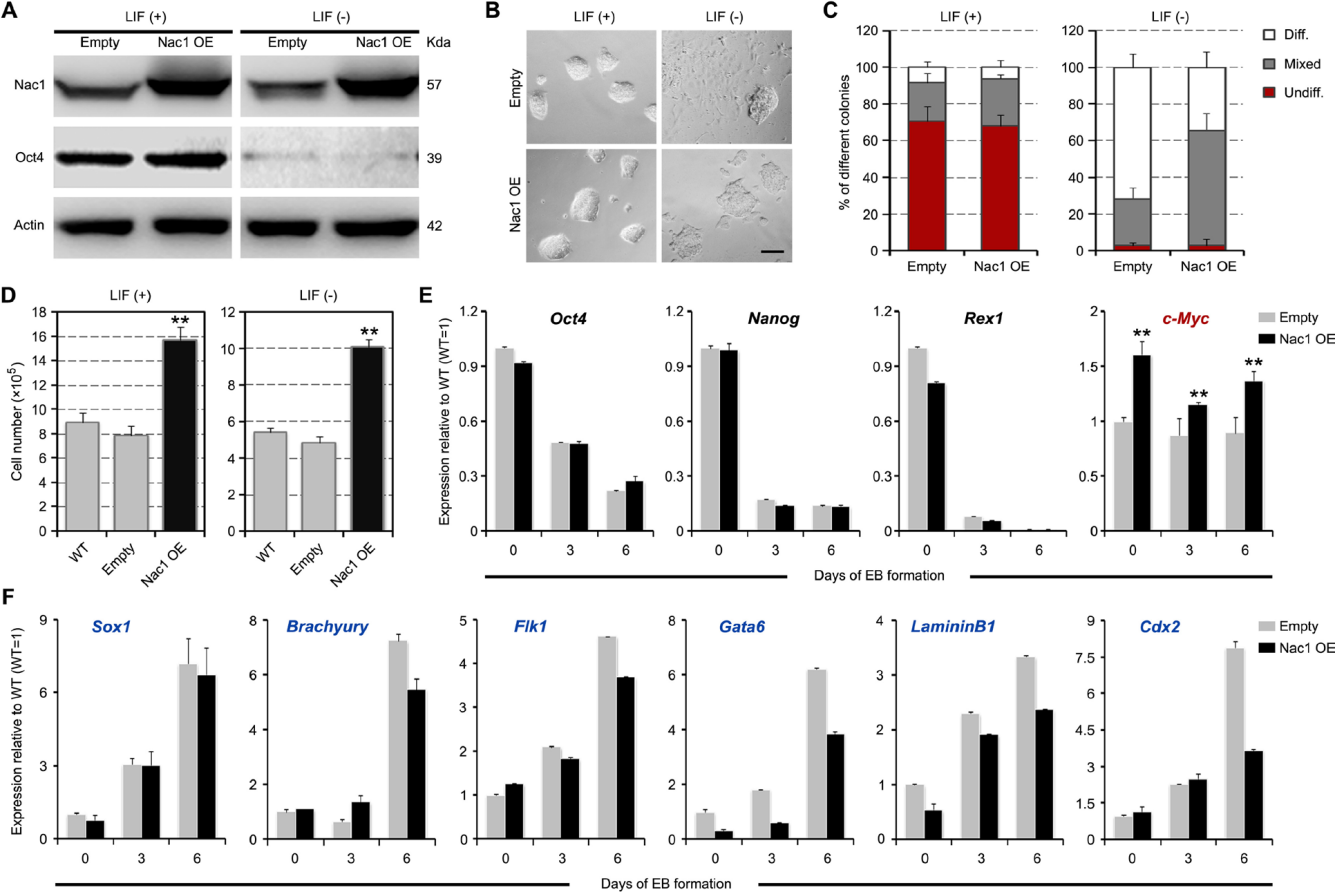
Nac1 OE delays but cannot block ESC differentiation (**A**) Immunoblot analysis after Nac1 OE. (**B**) Morphology of colonies formed by the indicated lines. Cells were grown with or without LIF for 6 days. Scale bar, 100 μm. (**C**) AP staining of colonies formed by plating the indicated cells at clonal density and culturing for 6 days with or without LIF. Percentage of colony types (undifferentiated, mixed, or differentiated) formed by cells is shown. (**D**) The indicated cells were cultured for 5 days with or without LIF and cell numbers were counted. (**E**, **F**) qRT-PCR analyses of pluripotency (E) and germ layer (F) marker expression levels in the indicated lines after 0, 3 and 6 days of EB differentiation. All data are normalized to *Gapdh* and shown relative to WT ESCs (set at 1.0). Data in (C–F) are represented as means ± s.d.; *n* = 3. *p < 0.05; **p < 0.01. All *P*-values were calculated using Student's *t*-test.

To investigate the effect of Nac1 gain of function on ESC self-renewal and pluripotency, full-length Nac1 cDNA was cloned into the pPyCAGIP-based vector and stable Nac1 OE ESC lines were established (Figure 4A). Under the +LIF conditions, there were no obvious differences in cell morphology and self-renewal colony formation ratio between the Nac1 OE and control cells (empty vector-transfected). However, under –LIF culture conditions, delayed morphological differentiation was observed in Nac1 OE cells (Figure 4B, 4C). In addition, Nac1 OE resulted in a significant increase in growth rate under both conditions (Figure 4D). These data suggest that Nac1 may enhance self-renewal and promote proliferation of ESCs. During EB formation, Nac1 OE cells showed a similar dynamic change of the core pluripotency genes but an increased expression of *c-Myc* when compared with control cells (Figure 4E). These results together with above data suggest that c-Myc may be a downstream effector of Nac1. Moreover, although the expression of all three germ layer markers were induced, Nac1 OE cells exhibited a delayed upregulation of these markers (Figure 4F), indicating that Nac1 OE delays (but does not prevent) differentiation of ESCs.

### Nac1 directly regulates *c-Myc* transcription

**Figure 5:**
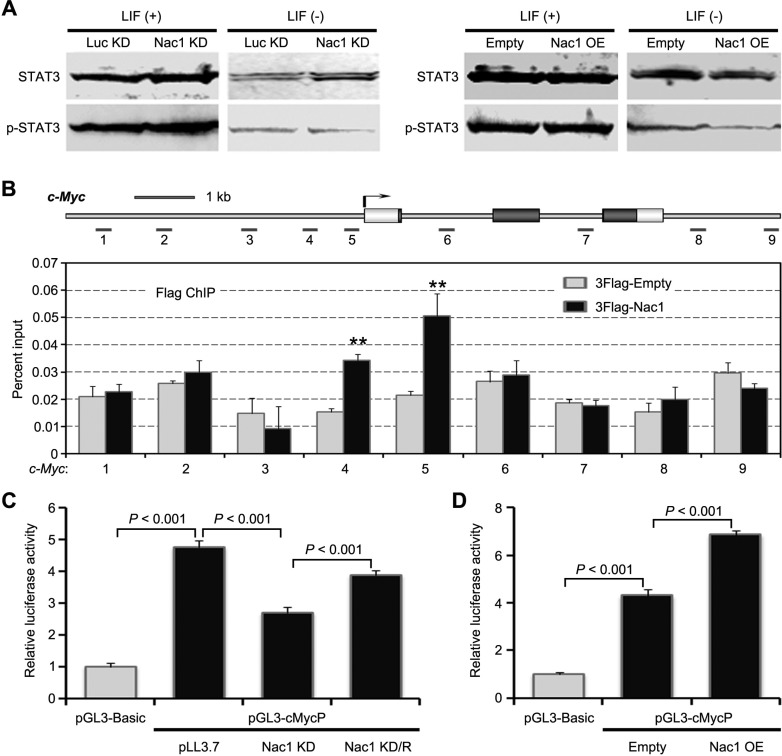
Nac1 directly binds and regulates c-Myc transcription (**A**) Immunoblot analysis of phospho-STAT3 and total STAT3 in the indicated cells cultured with or without LIF. (**B**) ChIP-qPCR analysis of Nac1 occupancy at *c-Myc* locus. Numbered grey bars indicate primer locations (upper). ESCs were transfected with the 3Flag-tagged Nac1 expression vector or the 3Flag-empty vector as a control. ChIP was performed using anti-Flag antibody and qPCR analysis was performed with the primers indicated. Values are expressed as percent of input DNA (lower). (**C**) Luciferase reporter analysis. The c-Myc promoter reporter-construct, pGL3-cMycP, spanned positions **−**2520 to +525 relative to the transcriptional start site, was transfected into the indicated ESC lines using Lipofectamine^2000^ with pRL-SV40 as an internal control. Transfected cells were cultured in the presence of LIF for 48 hr and then luciferase activity was measured. Values are normalized to a Renilla luciferase control. The mean value of ESCs transfected with the pGL3-Basic reporter vector was set at 1.0. Nac1 KD/R: Nac1 KD ESCs transfected by Nac1 OE vector. (**D**) pGL3-cMycP was transfected into the indicated ESC lines. Transfected cells were cultured in the presence of LIF for 48 hr and then luciferase activity was measured. Values are normalized to a Renilla luciferase control. The mean value of ESCs transfected with the pGL3-Basic reporter vector was set at 1.0. Data in (B–D) are represented as means ± s.d.; *n* = 3. *p < 0.05; **p < 0.01. All *P*-values were calculated using Student's *t*-test.

c-Myc has been reported to play an important role in regulating the proliferation and differentiation of ESCs [35, 36]. Based on the afore-mentioned results, we reasoned that Nac1 might regulate transcription of *c-Myc*. To this end, we examined whether Nac1 regulates STAT3 activity, which has been shown to directly control *c-Myc* transcription in ESCs. WB analysis indicated that STAT3 activation was not affected by either Nac1 KD or Nac1 OE in the presence or absence of LIF (Figure 5A). Next, we performed chromatin immunoprecipitation (ChIP) to assess whether Nac1 binds to the c-Myc locus (spanning from ~5 kb upstream of the transcription start site to 2 kb downstream of 3′ UTR) *in vivo*. Significantly, an enhanced enrichment of Nac1 on the *c-Myc* proximal promoter was observed (Figure 5B). To determine whether the binding was associated with the regulation of *c-Myc* transcription, we performed a luciferase reporter assay. We found that *c-Myc* promoter activity was repressed following Nac1 KD and increased following Nac1 OE (Figure 5C, 5D). Moreover, the decreased activity of *c-Myc* promoter by Nac1 KD could be rescued by re-expression of Nac1 (Figure 5C), suggesting that this effect is specific.

### Nac1 KD ESC phenotypes can be partially rescued by c-Myc restoration

**Figure 6:**
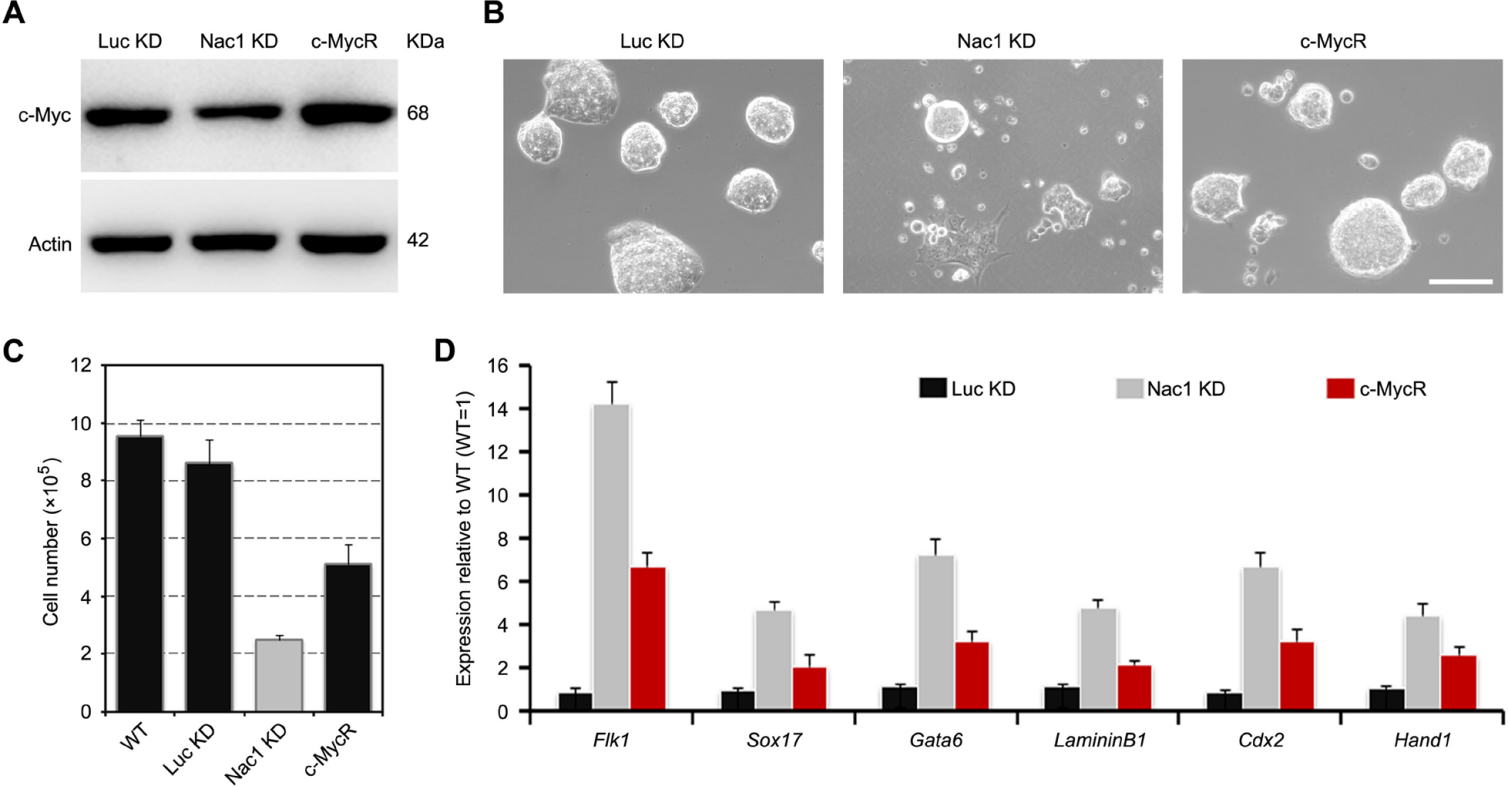
Restoration of c-Myc partially rescues Nac1 KD ESC phenotypes (**A**) Immunoblot analysis of Nac1 KD cells transduced with c-Myc + M2rtTA for 5 days. Cells infected with c-Myc DOX-inducible lentivirus + M2rtTA restore normal c-Myc expression levels upon addition of doxycycline (DOX). (**B**) Morphology of colonies formed by the indicated lines. Cells were grown with LIF and doxycycline for 5 days. Scale bar, 100 μm. (**C**) The indicated cells were cultured with LIF and doxycycline for 5 days and cell numbers were counted. (**D**) qRT-PCR analyses of germ layer marker expression levels in the indicated cells cultured with LIF and doxycycline for 5 days. All data are normalized to *Gapdh* and shown relative to WT ESCs (set at 1.0). Data are represented as means ± s.d.; *n* = 3.

We then asked whether restoration of c-Myc expression to normal levels in Nac1 KD cells had any biological effects. To avoid the possibility of neomorphic effects, we used a drug-inducible lentiviral system, which allows c-Myc to be expressed at nearly endogenous levels under the control of doxycycline [37] (Figure 6A). In contrast to the differentiation-like morphology induced by Nac1 KD, the c-Myc rescued (c-MycR) cells formed more compact dome-shaped colonies when cultured with LIF (Figure 6B). Cell growth rate and qRT-PCR analyses showed that the decreased proliferation ability and the upregulation of differentiation markers induced by Nac1 KD were partially rescued by restoration of c-Myc expression (Figure 6C, 6D). These results indicate that c-Myc is a downstream effector of Nac1 and partially mediated its functions in regulating ESC proliferation and self-renewal.

## DISCUSSION

Nac1, which is encoded by *Nacc1*, belongs to the Pox virus and Zinc finger/Bric-a-brac Tramtrack Broad complex (POZ/BTB) family. The N-terminus of Nac1 contains a conserved BTB domain which mediates protein-protein interactions [38, 39] and the C-terminus of this protein contains a putative BEN domain which may be responsible for DNA-binding activity [40]. It has been reported that Nac1 is involved in transcriptional regulation, ubiquitin proteasome-mediated protein degradation, cell proliferation, apoptosis and autophagy [41–46]. Many previous studies have focused on the role that this protein plays in psychomotor stimulant response [47–49] and cancer aggressiveness [26, 29, 46]. In ESCs, Nac1 has been identified as a protein-interacting partner of Nanog and Oct4 [30, 31], but little is known about the function and molecular mechanism of Nac1 in regulating self-renewal and pluripotency.

In this study, we observed that Nac1 OE promoted ESC proliferation and delayed ESC differentiation. By contrast, Nac1 KD inhibited ESC proliferation and upregulated the expression of differentiation markers. Nonetheless, Nac1 KD ESCs maintained the normal expression levels for pluripotency genes, and could be induced to differentiate into all three germ layers. Therefore, we conclude that Nac1 is a self-renewal promoting factor, but is not essential for sustaining pluripotency of ESCs. These results provide a reasonable explanation for why Nac1 knockout mice suffer from a survival disadvantage, but show almost normal development except for vertebral patterning defect [32].

Recently, Nac1 was reported to be needed for ME differentiation of ESCs. Nac1 KD resulted in a significant reduction of the ME fate, but induced the NE fate choice [33]. However, our study indicates that Nac1 KD facilitates the ME differentiation of ESCs. This discrepancy may be due to the different approaches utilized for gene knockdown and ESC differentiation. In Malleshaiah *et al*.'s study, siRNAs targeting Nac1 were transiently transfected into ESCs, and the immunostaining for Brachyury (T) expression at 72 hr of differentiation was the only phenotypic evaluation performed [33]. As part of our analysis, we established stable Nac1 KD ESC lines and induced EB differentiation to mimic the temporal and spatial processes of the developing embryo. In accordance with the study performed by Malleshaiah *et al*., we observed that there were no obvious expression changes of *Brachyury* in Nac1 KD ESCs and early EBs. However, the other ME markers, such as *Flk1*, *Gata6*, *LamininB1* and *Sox17*, were all upregulated by Nac1 KD. Moreover, these genes, including *Brachyury*, were significantly further upregulated in day 6 EBs derived from Nac1 KD ESCs. These results are also consistent with the observations of Wang *et al*. [31]. Brachyury has been reported to be an important regulator of early mesoderm development [50, 51]. Our data implicate that Nac1 KD cells might differentiate to ME through a Brachyury independent pathway.

Maintenance of the pluripotency state of ESCs depends on both the power of self-renewal and the capacity to block differentiation [16]. Currently, several lines of evidence indicate that Nac1 may directly repress the transcription of developmental genes. First, Nac1 has been found to be highly enriched at the promoter regions of development-associated genes [17, 31, 33]. Second, Nac1 deficiency induces the upregulation of developmental genes without reducing the expression of core pluripotency factors. Third, the upregulated developmental genes in Nac1 KD cells can only be partially rescued by c-Myc. However, Nac1 OE cannot block the upregulation of differentiation markers under –LIF conditions, suggesting that the inhibitory actions of Nac1 on the transcription of development-associated genes are either cell context-dependent or required to cooperate with other stemness factors.

Based on genome wide protein-protein and protein-DNA interaction analysis, Nac1 has been identified as a core module pluripotency-associated TF [17, 30, 31]. However, unlike the other core stemness factors that are expressed almost exclusively in pluripotent stem cells, Nac1 is widely distributed in adult tissues [52]. Our expression pattern analysis also shows that cell type-specific expression of Nac1 does not occur at the early stages of embryo development. After EB differentiation, Nac1 expression decreases very slowly, whereas the other core module pluripotency TFs are rapidly downregulated. More importantly, Nac1 KD or OE has no significant effect on these factors. Thus, these core factors are unlikely to be downstream effectors of Nac1, and vice versa.

In addition to the core stemness factors, c-Myc is another key player that is crucially involved in maintaining the pluripotency and self-renewal of ESCs [53]. Although c-Myc and N-myc are functionally redundant [36], c-Myc deficiency has been proved to antagonize self-renewal and promote differentiation. In contrast, c-Myc overexpression confers LIF independent self-renewal on ESCs [54]. In this study, we provide direct evidence that Nac1 regulates c-Myc transcription. We also demonstrate that the function of Nac1 in promoting ESC self-renewal is at least partly mediated by c-Myc. However, Nac1 OE failed to completely prevent ESC differentiation, despite increased expression of c-Myc. This result may be due to the fact that upregulated c-Myc expression is not sufficient to fully inhibit the activation of developmental genes under differentiation culture conditions. Conversely, c-Myc re-expression appears to only partially rescue the reduced self-renewal observed for Nac1 KD cells, indicating that other mechanisms might be responsible for Nac1 activity in ESCs.

ESCs fluctuate between the naive/ground state (with enhanced self-renewal efficiency and robust pluripotency) and the primed state (with increased differentiation propensity) [55, 56]. Most recently, c-Myc was reported to play a critical role in controlling the biosynthetic machinery of ground state naive ESCs without affecting their potency [57]. c-Myc depleted stem cells enter a state of dormancy similar to embryonic diapauses [35]. Our data support these findings by revealing that the Nac1/c-Myc axis is essential for the proliferation and self-renewal of ESCs but dispensable for preserving pluripotency. However, to what extent and how this mechanism of action contributes to control ESC status require further elucidation.

In ESCs, it has been shown that the core and c-Myc-centered modules are functionally separable [18]. However, this thesis is predominantly based on physical bindings of protein and DNA, rather than functional studies. Our data reveal that there is a transcriptional regulatory relationship between Nac1 and c-Myc. This suggests a functional link between the core and c-Myc-centered networks in ESCs. It should be also noted that ESCs and cancer cells both exhibit c-Myc module activity [18]. Both Nac1 and c-Myc are oncogenes [45, 58]. To the best of our knowledge, up until now, no report has been published describing the association between Nac1 and c-Myc in cancer. In the past decades, intensive researches have revealed that deregulated c-myc expression plays a central role in human tumorigenesis and the c-Myc represents a promising target in anticancer therapy. However, the mechanisms for deregulation of c-myc in cancer are still not fully known. Therefore, our findings provide a new insight into the putative mechanisms that underlie tumor initiation and development.

## MATERIALS AND METHODS

### Plasmid construction

TetO-FUW-cMYC was a gift from Rudolf Jaenisch (Addgene plasmid # 20324). The pLL3.7 vector (provided by Luk Van Parijs) was modified by replacing the EGFP gene with the zeocin resistance gene. All shRNA targeting sequences (Supplementary Table 1) were designed and BLASTed to ensure specificity. The oligonucleotides encoding target shRNA were cloned as described before [59]. The pPyCAGIP expression vector was a gift from Ian Chambers. The full-length ORF of Nac1 was PCR-amplified from mouse ESC cDNA using KOD-Plus- (TOYOBO). The amplified ORF was subsequently cloned into pGEM-T Easy (Promega) for sequence verification, and then subcloned into pPyCAGIP. The 3×FLAG fragments were generated from the p3×FLAG-CMV plasmid via PCR, and subcloned in-frame into the expression vectors. The *c-Myc* promoter (positions **−**2520 to +525) was amplified by PCR from mouse genomic DNA and inserted into pGL3-basic vector (Promega). The primers for construction are listed in Supplementary Table 2.

### Cell culture

293FT cells (Invitrogen), mouse ESC lines R1 (American Type Culture Collection), CCE (Stemcell Technologies) and their derivatives were cultured as described before [59]. For EB differentiation, ESCs were trypsinized to single-cell suspensions, plated at a density of 5 × 10^4^ cells/ml in Petri dishes and cultured in LIF-deficient ESC medium for the indicated number of days. The medium was changed every 2 days.

### ESC transfection, lentiviral production and infection

The plasmid DNA was transfected using Lipofectamine 2000 (Invitrogen) according to the manufacturer's instructions. For stable transfection, resistant colonies were pooled and expanded for further analysis. Lentiviral production and infection were performed as previously reported [59]. In brief, the 293FT cells were transfected with the lentiviral vector, pSPAX2 and pMD2G. ESCs were trypsinized and infected in suspension by the viral supernatant along with polybrene (4 μg/ml; Sigma). Cells were then plated and subjected to selection for resistance to antibiotics.

### Cell proliferation and colony formation assay

For the proliferation assay, cells were plated at a density of 1000 cells/cm^2^ in gelatin-coated dishes and cultured in the presence or absence of LIF. Cells were counted after 5 days in culture. Viable cells were determined by Trypan blue exclusion and manually counted with a hemocytometer under light microscopy. For the colony formation assay, ESCs were plated at clonal density (200 cells/cm^2^) and cultured in the presence (1 ng/ml) or absence of LIF. After 6 days, cells were subjected to AP staining (Millipore) according to the manufacturer's instructions. Colonies were scored in three categories: undifferentiated, mixed (partially differentiated) and differentiated.

### Mouse embryo collection and immunofluorescence

Pre-implantation embryos were flushed out from the oviducts or uterus, fixed in 4% formaldehyde for 20 min at 4°C and permeabilized with 0.1% Triton X-100 for 15 min. This was followed by blocking with 1% bovine serum albumin (BSA)/phosphate-buffered saline (PBS) for 30 min. Immunofluorescence staining for whole embryos was carried out as previously described [59]. A list of antibodies and dilution ratios is available in the Supplementary Table 3. Cell nuclei were visualized by staining with DAPI. Images were captured with a confocal microscope (LSM710, Zeiss).

### Immunoblotting and real-time PCR (qRT -PCR) analyses

Western blot and qRT-PCR procedures were described previously [59]. The antibodies and qRT-PCR primers used in this study are listed in Supplementary Tables 3 and 4 respectively.

### Luciferase assay

Briefly, 0.5 μg of luciferase reporter was co-transfected into ESCs using Lipofectamine 2000 with 0.01 μg of pRL-SV40 (Promega) as an internal control. Transfectants were lysed at 48 h after transfection and luciferase activities were measured using the Dual-luciferase Reporter Assay System (Promega).

### Chromatin immunoprecipitation (ChIP)

ChIP was performed using the ChIP Assay Kit (Upstate Biotechnology) according to the manufacturer's instructions. Briefly, cells were cross-linked with 1% formaldehyde for 10 min at room temperature, and quenched using 125 mM glycine. Chromatin extracts containing DNA fragments with an average size of 500 bp were immunoprecipitated using Flag antibody (Beyotime). For all ChIP experiments, qPCR analyses were performed using the Eco real-time PCR System (Illumina) and SYBR green master mix, as described before. Enrichment was calculated relative to input. The ChIP-qPCR primers are listed in Supplementary Table 5.

### Statistical analysis

Statistical analysis was performed using the Statistical Package for Social Science (SPSS for Windows package release 13.0; SPSS Inc., Chicago, IL). The Student's *t*-test was used to analyze statistical differences. Data in the figures were expressed as mean ± SD or mean ± SEM, and p < 0.05 was considered significant. Each experiment was performed at least three times.

## SUPPLEMENTARY MATERIALS TABLES


