# Supplementary material for: Nac1 promotes self-renewal of embryonic stem cells through direct transcriptional regulation of c-Myc
Source: Oncotarget. 2017 May 10;8(29):47607–18. doi: 10.18632/oncotarget.17744 (PMC5564591; doi:10.18632/oncotarget.17744)
Supplement: Supplementary file 1 [file oncotarget-08-47607-s001.pdf]

Nac1 promotes self-renewal of embryonic stem cells through direct transcriptional regulation of c-Myc

Supplementary Materials

Supplementary Table 1: Target sequences of shRNA against target genes

Name	Target Gene	Sequence (sense strand)	Starting Nucleotide from ATG (bp)
Nac1 KD-1	Nacc1 (CDS)	GATGAGCAGTACCGTCAGATC	838
Nac1 KD-2	Nacc1 (3′ UTR)	GGTTTCCAGTCTGGCAGAA	2483
Nac1 KD-3	Nacc1 (3′ UTR)	GCACAAGCACCTTAGTCATA	3406
Nac1 KD-4	Nacc1 (3′ UTR)	GTGAGATTGCACATTAACTACTGTA	4005
Luc KD	Luciferase	GACGAACACTTCTTCATCG	1285

Supplementary Table 2: Primers for plasmid construction

Name	Primer Sequence (5′–3′)	
	Forward	Reverse
pPyCAGIP-mNac1	CGGAATTCGGATCCACCATGGCGCAGACCTTAC	CGGGATCCGAATTCTTACTGGAGAACCTCA
pGL3-cMycP	ACTGTGTGAGTTTCAGGCTAGCA	AGCCTTCAAACAGCTCGAGGAG

Supplementary Table 3: Antibody details

	Antibody	Source	Cat. no.	Dilution
**Primary**	Nac1	Abcam	ab-151426	1:1000 (WB)
	Oct4	Santa Cruz	sc-5279	1:100 (IF, FCM); 1:1000 (WB)
	Cdx2	BioGenex	MU392A-UC	1:100 (IF, FCM)
	STAT3	eBioscience	14-6013-81	1:1000 (WB)
	Phospho-STAT3	cell signaling	#9131	1:1000 (WB)
	c-Myc	Santa Cruz	sc-40	1:750 (WB)
	β-Actin	Sigma	A1978	1:2000 (WB)
	FLAG	Beyotime	AF519	1:200 (ChIP); 1:1000 (WB)
**Secondary**	Donkey anti-Rabbit IgG (H&L), DyLight®650 Conjugate	ImmunoReagents	DkxRb-003-E650NHSX	1:200 (IF, FCM)
	Donkey anti-Goat IgG (H&L), DyLight®594 Conjugate		DkxGt-003-E594NHSX	
	Donkey anti-Mouse IgG (H&L), DyLight®488 Conjugate		DkxMu-003-D488NHSX	
	Goat anti-Mouse IgG/HRP	zhongshan	ZDR-5307	1:4000 (WB)
	Goat anti-Rabbit IgG/HRP		ZDR-5306	

Supplementary Table 4: Primers for qRT-PCR analysis

Gene	Primer Sequence (5′–3′)	
	Forward	Reverse
GAPDH	AGGTCGGTGTGAACGGATTTG	TGTAGACCATGTAGTTGAGGTCA
Oct4	CACGAGTGGAAAGCAACTCA	AGATGGTGGTCTGGCTGAAC
Nanog	TCTTCCTGGTCCCCACAGTTT	GCAAGAATAGTTCTCGGGATGAA
c-myc	TCACCAGCACAACTACGCCG	CAGGATGTAGGCGGTGGCTT
Klf4	CTAAATGATGGTGCTTGGTGA	TGGCTTAGGTCATCAATGTAG
Rex1	CCCCAAATACCACTGACCAA	AACTCACCTCGTATGATGCA
Sox1	TGAACGCCTTCATGGTGTGGTC	GCGCGGCCGGTACTTGTAAT
Nestin	AGATCGCTCAGATCCTGGAA	AGGTGTCTGCAAGCGAGAGT
Brachyury	CTGCGCTTCAAGGAGCTAAC	CCAGGCCTGACACATTTACC
Flk1	TTTGGCAAATACAACCCTTCAGA	GCAGAAGATACTGTCACCACC
Sox17	GTGGACCGCACGGAATTCGAA	GCAATAGTAGACCGCTGAGCTA
Gata6	AAAGCTTGCTCCGGTAACAG	TTCTCCCACTGCAGACATCA
LamininB1	GAAAGGAAGACCCGAAGAAAAGA	CCATAGGGCTAGGACACCAAA
Cdx2	CAAGGACGTGAGCATGTATCC	GTAACCACCGTAGTCCGGGTA
Hand1	TGAACTCAAAAAGACGGATGG	CTTTAATCCTCTTCTCGCCG

Supplementary Table 5: Primers for ChIP-qPCR analysis

Name	Primer Sequence (5′–3′)	
	Forward	Reverse
pcMyc-1	GACATCTAACTCACATCCAGC	CCTCTGTGGACAAGCTTACTA
pcMyc-2	GCTCTACCCACTGTCATATC	TCTCTTAGAGGTTGCGCACA
pcMyc-3	AAGCATCTTCCCAGAACCTG	TTGTGGCTCTCGGATTTGTG
pcMyc-4	CACCCCAGCTCCTAAACCAG	ACACCTCCACACAGTTCCAG
pcMyc-5	ACCTGCGGTGACTGATATAC	GTCCTTTCCCTTTCTGTACG
pcMyc-6	GATGTGACCGATTCGTTGAC	CAGTCTTCCTAGCAATTCAG
pcMyc-7	GAAAGGTTCTCGGTAAAGTC	AATCAGATCCAGACACCATC
pcMyc-8	GCAGAGCTTAATGGGTACGA	ATTCTCAGACCCCTAACCAG
pcMyc-9	CTTCCAGAAAGTCAGGCTGA	CCCTCATCGTGATTGTTATC
